# Scope of Nursing Work and Models of Service Delivery in Australian Primary and Secondary Schools: A Scoping Review

**DOI:** 10.1111/jan.70562

**Published:** 2026-03-13

**Authors:** Anita Moyes, Chelsey Williams, Elizabeth Rankin, Imelda Hoodcamp, Eliza‐Jane Potter, Brent A. Hayward

**Affiliations:** ^1^ School of Nursing and Midwifery Edith Cowan University Perth Western Australia Australia; ^2^ South Australian School Nurses Association Adelaide South Australia Australia; ^3^ Calvary Christian College Queensland Australia; ^4^ Independent Researcher Central Coast New South Wales Australia; ^5^ School of Nursing and Midwifery, Monash University Clayton Victoria Australia

**Keywords:** Australia, children, school nursing, schools, scoping review, students

## Abstract

**Aim:**

To map the scope of nursing work and models of service delivery in Australian primary and secondary schools for children aged 3–18 years.

**Design:**

Scoping Review.

**Data Sources:**

A search of CINAHL, Medline, PsycINFO, ERIC, Informit and Google was conducted in August 2024 for peer reviewed, non‐peer reviewed and grey literature giving insight into nursing work in primary and secondary Australian schools in urban, regional and remote areas of all Australian states and territories.

**Methods:**

The review employed Johanna Briggs Institute methodology for scoping reviews and reported the findings in line with the Preferred Reporting Items for Systematic Reviews and Meta‐Analyses extension for Scoping Reviews (PRISMA‐ScR) Checklist.

**Results:**

One hundred and forty‐two sources were included. Findings indicate that nurses working in Australian schools conduct a wide range of activities which vary by jurisdiction, education sector, employer and school type. Models of nursing service delivery are similarly varied and range from full‐time school‐based nurses to nurses who visit schools on an occasional basis.

**Conclusion:**

The varied scope of nursing work and models of service delivery provide evidence that the nursing workforce in schools is adaptable and flexible, but unequal access to nursing services raises important questions about equity. There is an urgent need for a national approach to nursing work in Australian schools.

**Impact:**

This is the first review to map the scope of nursing work and models of service delivery in Australian primary and secondary schools.

## 1 Introduction

The World Health Organization (WHO) recommends comprehensive school health services in all countries (WHO 2021a). The most common health professionals working in schools are nurses, but the utility of nurses in schools varies both between and within countries (Baltag et al. 2015). Nurses have been working in Australian schools for over a century (Moyes 2021). While this history suggests a mature field of practice, the available evidence has never been synthesised. According to the national school nursing standards for practice, nurses working in Australian schools commonly assess and intervene with complex student health needs including physical health (chronic illness and acute presentations), mental and psychosocial health concerns, disability support and identification of developmental concerns (Australian Nursing and Midwifery Federation 2019). However, there is no national approach to school nursing or school health services, and how service provision varies across jurisdictions and employers remains unclear (Williams et al. 2024). The Australian federal government recently endorsed nurses in schools as a strategy for improving student wellbeing (Australian Government Department of Education 2024; Hayward and Moyes 2025). This development provides a strong impetus for more closely examining the work of nurses in Australian schools. This scoping review investigates the scope of nursing work and models of nursing service delivery in Australian schools, a topic that has not previously been investigated.

## 2 Background

Schools are an ideal setting for promoting health. In most countries, children and young people spend a significant amount of time in school, and the access to children and young people afforded by schools is unparalleled. The structured nature of the school environment facilitates health education, early identification of health and developmental concerns, as well as offering opportunities for intervention (Garvey et al. 2020). Importantly, children who are healthy are more likely to benefit from the educational opportunity schools provide, while education in turn facilitates academic achievement, a recognised social determinant of longer‐term health outcomes (Editorial 2020).

The opportunity to advance children and young people's health by capitalising on schools as a setting for health has a long history and was re‐affirmed in 2021 through the *Making every school a health‐promoting school* initiative (WHO and UNESCO 2021). The health‐promoting school initiative has six ‘pillars’: (i) healthy school policies; (ii) a healthy school physical environment; (iii) a healthy school social environment; (iv) health education and skills; (v) links with parents and the broader community, and (vi) access to school health services (WHO 2021b). While each of these is important, the first five pillars are primarily in the remit of education professionals such as teachers. The sixth pillar – access to school health services on school premises or in a location adjacent to the school – transforms the school from a solely educational institution to a practical site for the implementation of primary health care. Crucially, school health services are provided by a health worker, not an education professional. While Australia has localised examples of multidisciplinary, integrated school health services consistent with WHO recommendations (e.g., Rungan et al. 2024), Australia does not have a national approach to school health services or school nursing. This is despite Australian national standards for school nurses to guide practice (Australian Nursing and Midwifery Federation 2019) and a national plan for children's health (Department of Health 2019).

The fragmentation of school health services in Australia reflects the country's political system and its health and education funding arrangements. The Commonwealth of Australia is comprised of six states and two territories. School funding is shared between the Commonwealth and state/territory governments, with states and territories contributing the majority of funding to the public sector and the Commonwealth to the non‐government sector (Australian Government Department of Education 2025). States and territories interpret national laws and policies, subsequently developing their own policies, funding models, and reform agendas for implementation by individual schools (Boyle and Anderson 2020). This complexity extends to the health sector, where states and territories manage hospitals, health institutions and health services, while the Commonwealth government oversees national health policy, and provides funding to states and territories for public hospital services and population‐specific services (Australian Government Department of Health, Disability and Ageing 2025). Both levels of government share responsibility for health workforces, healthcare quality, and responding to national emergencies (Waller et al. 2021). Nurses working in schools can be employed by education or health departments or directly by a school (Williams et al. 2024). This employment structure has led to a heterogenous approach to nursing work in Australian schools at a time when student health needs are intensifying.

Australian children and young people are coming to school with increasingly complex health issues. The prevalence of chronic conditions has doubled over the last two decades (Hu et al. 2022), with close to 50% of children and adolescents managing one or more chronic health conditions (Australian Institute of Health and Welfare 2024a; Osman et al. 2025). One in five Australian children has additional health and developmental needs at school entry (Garvey et al. 2020), and inclusive education policies across states and territories promote the full participation and learning of all children, regardless of their health or developmental status (Boyle and Anderson 2020). The additional responsibility on school staff to meet these needs is so high that Australian researchers have suggested that teachers have become quasi health workers (Rossi et al. 2016). This additional burden on teachers (Marks et al. 2018), at a time when Australian teacher wellbeing is already low (Carroll et al. 2022), underscores a need for a more comprehensive approach to student health in Australian schools.

Recognising these pressures, the Australian Commonwealth government has taken a stronger interest in health service provision at school, specifically identifying nurses as potential contributors to addressing this issue (Australian Government Department of Education 2024; Hayward and Moyes 2025). While the long history of school nursing in Australia suggests a mature field of practice, what nurses do in schools and how these services are delivered is poorly understood (Moyes and Hayward 2025; Moyes et al. 2024). Available evidence is fragmented, not cumulative, having never been synthesised. Understanding the scope of nursing work and models of service delivery in Australian schools is a necessary prerequisite for developing research programs that can meaningfully examine school nursing's impact on student outcomes. A scoping review synthesising the evidence is therefore an essential first step. The aim of this scoping review is to collate, synthesise and summarise the evidence to map the scope of nursing work and models of service delivery in Australian schools. The review seeks to answer the question: What is the scope of nursing work and models of service delivery in Australian primary and secondary schools for children aged 3–18 years, and how does this vary by state or territory and education sector?

## 3 Methods

### 3.1 Design

The review was conducted according to JBI methodology for scoping reviews (Peters et al. 2020) and reporting was guided by the Preferred Reporting Items for Systematic Reviews and Meta‐Analyses extension for Scoping Reviews (PRISMA‐ScR; Page et al. 2021). The protocol was published at https://doi.org/10.11124/JBIES‐24‐00151 and the review pre‐registered at https://osf.io/6yqrm.

### 3.2 Inclusion and Exclusion Criteria

Peer reviewed, non‐peer reviewed and grey literature addressing the review question, unrestricted by date or language, were included. Protocols, editorials, conference papers, commentaries and papers with the sole focus of advocating for nursing work in schools were excluded. Only static documents such as published reports were included. Changeable sources of information such as Government websites were excluded.

### 3.3 Participants

The review included studies of nurses providing any type of health service in a primary or secondary Australian school. Dental nurses were excluded due to their distinct scope of practice, unique educational preparation, regulatory framework and professional governance structures.

### 3.4 Concept

The review focused on two concepts: scope of nursing work and models of nursing service delivery. Scope encompassed documented nursing activities, such as immunisation, sports injury management, or vision screening. Models of nursing service delivery identified the frequency with which the nurse was present in the school.

### 3.5 Context

All Australian primary and secondary schools, both government and non‐government, providing education to children aged 3–18 years were included. Childcare settings and standalone pre‐school settings such as community kindergartens were excluded as these fall outside the primary and secondary school context. Post‐secondary education settings such as universities and technical colleges were also excluded.

### 3.6 Search Strategy

The systematic search was conducted in three phases (Peters et al. 2020). Initially, CINAHL and Medline were used to identify relevant keywords and index terms. This informed the search strategy, which was reviewed by an academic librarian. Subsequently, comprehensive searches, unrestricted by date or language, were performed in CINAHL, Medline, PsycINFO, and ERIC, all via EBSCOHost and Informit via the native interfaces, using database‐specific adaptations of the search strategy. The search strategy for CINAHL is shown in Table 1 as an example. Grey literature was identified by searching the first five pages of Google (Bonato 2018). Reference lists of included articles were also manually reviewed for additional sources. Finally, a small number of ‘other sources’ were identified by team members with expert knowledge of school nursing in Australia.

**TABLE 1:** CINAHL search strategy.

Search	Query	Records retrieved
#1	TI school* OR AB school* OR TI school‐based OR AB school‐based	175,062
#2	TI nurs* OR AB nurs*	616,528
#3	TI Australia* OR TI Queensland* OR TI New South Wales OR TI Australian Capital Territory OR TI Victoria* OR TI Tasmania* OR TI South Australia* OR TI West* Australia* OR TI Northern Territory	52,351
#4	AB Australia* OR AB Queensland* OR AB New South Wales OR AB Australian Capital Territory OR AB Victoria* OR AB Tasmania* OR AB South Australia* OR AB West* Australia* OR AB Northern Territory	71,125
#5	S3 OR S4	98,666
#6	S1 AND S2 AND S5	407
#7	((MH ‘School Nursing’) OR (MH ‘School Nurses’))	10,406
#8	(MH Australia OR MH Queensland OR MH New South Wales OR MH Australian Capital Territory OR MH Victoria OR MH Tasmania OR MH South Australia OR MH Western Australia OR MH Northern Territory)	133,040
#9	S7 AND S8	93
#10	S6 OR S9	473

Sources were imported into Rayyan (Ouzzani et al. 2016) for duplicate removal and screening. Two authors independently conducted pilot screenings of 25 randomly selected titles, abstracts and their full texts, discussing discrepancies until achieving > 75% inter‐rater agreement. All titles and abstracts were subsequently screened independently by at least two authors, and full‐text reviews were undertaken to confirm source eligibility. Disagreements were resolved through discussion with a third member of the team.

### 3.7 Study Selection

**FIGURE 1:**
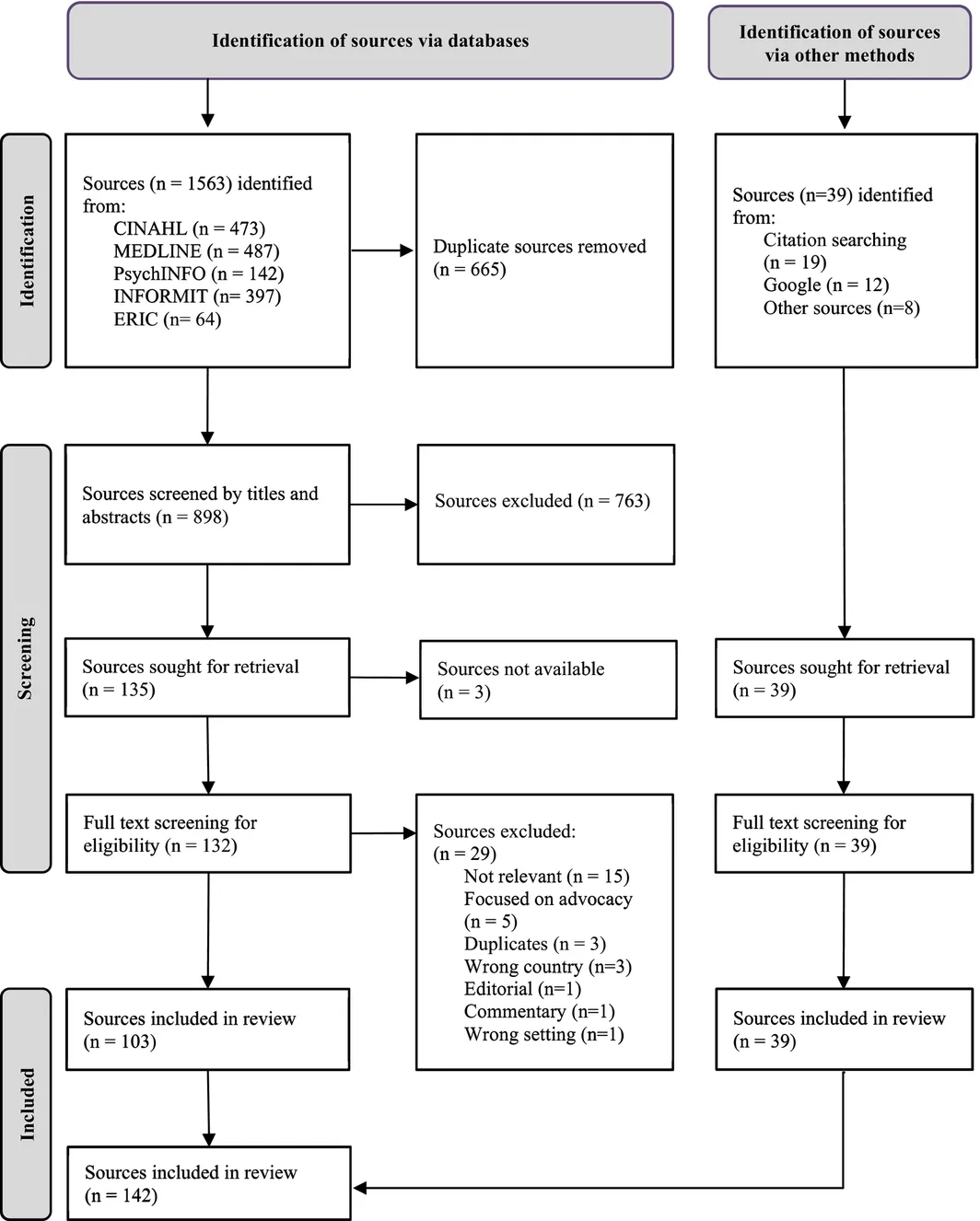
PRISMA flow diagram.

The systematic search returned 1563 sources of which 665 were duplicates and removed. The remaining 898 sources were screened by title and abstract, and a further 763 sources were excluded. The full texts of the remaining sources (135) were sought for retrieval; 3 could not be retrieved as they were unavailable. The remaining 132 sources underwent full text screening to confirm eligibility, resulting in the exclusion of a further 29 sources. Reasons for exclusion are shown in the PRISMA flow chart (Figure 1). The final number of sources included from the systematic search was 103. Citation searching (*n* = 19), Google searches (*n* = 12) and sources identified by team members (*n* = 8) identified a further 39 sources; therefore, a total of 142 sources were included in the review. The search results are illustrated in the PRISMA flow diagram (Figure 1).

### 3.8 Data Extraction and Charting

#### 3.8.1 Process

Data extraction was conducted independently by two authors using a tool adapted from the JBI template and consistent with JBI recommendations (Peters et al. 2020), and shown in Table 2. The tool was pilot tested to verify its feasibility and alignment with the review objectives. The extracted data included title, author/s, publication year, study type and study aim (for peer‐reviewed papers only), geographical context (Australian state/territory, urban/regional/remote setting), education sector (primary/secondary/both, government/non‐government schools) and service delivery characteristics (employer, nurse's occupational title, scope of work, service delivery model). The tool also recorded whether papers were part of a larger program of research or standalone studies, and their peer‐review status. Where data was missing, entries were marked ’unspecified’.

**TABLE 2:** Data extraction tool.

Item	Description
Title	The title of the source
Authors	The listed authors of the source
Year	Year of publication
Aims	The aim of study as stated by the authors (peer reviewed literature only).
Study type	The study design, methodology or methods as described by the authors (peer reviewed literature only).
State	The Australian state or territory in which the study was conducted.
Urban/regional/remote	Whether the paper reflects an urban, regional or remote school setting.
Primary/secondary/other school type	Whether the paper relates to primary school students, secondary school students, or a mixed cohort of students.
Government/non‐government school	Whether the paper describes school nursing services provided in government schools, non‐government schools or both.
Nurse employer	Whether the entity employing the nurse is the government or the school. Where the employer is the government, whether the employer is the Department of Health or the Department of Education.
Occupational title	The occupational title for the nurse working in a school as stated by the authors.
Scope of work	The scope of nursing work described.
Model of service delivery	How nursing services are delivered, including whether the nurse is based in or visits the school; whether they work autonomously or as part of a team. Where available, additional information such as the hours of nursing service availability may be included.
Service/program identifier	Identifies whether the paper is part of a suite of papers describing the same service/program, or whether the paper is a single study.
Peer Review (Y/N)	Whether the paper was peer reviewed or not peer reviewed.

#### 3.8.2 Categorisation

Initial analysis revealed that the scope of nursing work and models of service delivery could be categorised according to common characteristics. Given the high number of included sources and the enhanced potential to compare the scope of nursing work and models of service delivery between states and territories using descriptive statistical methods, this offered the opportunity to report the findings as frequencies and percentages in place of a narrative synthesis. Although pragmatically justified, a limitation of this approach is that frequency counts may reflect publication patterns, not true service prevalence.

### 3.9 Scope of Nursing Work

The scope of nursing work was categorised according to the World Health Organisation's menu of interventions for school health services (WHO 2021a) as shown in Table 3. This framework provides a structured approach to analysing school health service delivery through two key dimensions: health areas and types of health activities undertaken. The dimension ‘health areas’ encompasses eight domains in school health: positive health and development; unintentional injury; violence; sexual and reproductive health; communicable diseases; noncommunicable diseases (including sensory functions, physical disability, oral health, nutrition and physical activity); and mental health, substance use, and self‐harm. The eighth domain (general/cross‐cutting health issues) categorises interventions that do not fit neatly into the other seven domains. The dimension ‘activity types’ describe seven distinct approaches to school health service delivery: health promotion; health education; screening leading to care and/or referral; preventive interventions; clinical assessment leading to care and/or referral; health services management; and support for other pillars of health‐promoting schools (WHO 2021a). Included sources were categorised in as many domains as was supported by the evidence.

**TABLE 3:** Heat map of frequency of nursing activities categorised by the World Health Organisation's (2021a) menu of interventions for school health services.

		Type of SHS activity						
		1. Health promotion	2. Health education	3. Screening leading to care and/or referral	4. Preventive interventions	5. Clinical assessment leading to care, support and/or referral	6. Health services management	7. Support for other pillars of health‐promoting schools
Health area	a. general and cross‐cutting health issues	a1. promotion of care seeking; promotion of health literacy	a2. support for health promoting curriculum, including delivery of health education in the classroom	a3. school entry health check‐ups	[WHO menu blank]	a5. provision of first aid; administration of medications; referral and support for minor illness	a6. use data on school health services for monitoring and improvement	a7. support for policies on health promotion, injury prevention and other aspects of health‐promoting schools; training of school staff
	b. positive health and development	b1. promotion of appropriate use of electronic devices, adequate sleep, and parenting skills	[WHO menu blank]	[WHO menu blank]	[WHO menu blank]	b5. counselling and support for positive health, development and wellbeing	[WHO menu blank]	[WHO menu blank]
	c. unintentional injury	[WHO menu blank]	c2. provision of education to prevent unintentional injury	[WHO menu blank]	[WHO menu blank]	c5. medical management of injury beyond first aid	[WHO menu blank]	[WHO menu blank]
	d. violence	[WHO menu blank]	d2. provision of education to prevent violence	[WHO menu blank]	[WHO menu blank]	d5. support and referral for victims of violence (child abuse, neglect)	[WHO menu blank]	[WHO menu blank]
	e. sexual and reproductive health	e1. promotion of menstrual hygiene management	e2. provision of sexual and reproductive health education	[WHO menu blank]	[WHO menu blank]	e5. counselling and referral for STIs, contraception, and pregnancy	[WHO menu blank]	[WHO menu blank]
	f. communicable diseases	f1. promotion of personal hygiene	[WHO menu blank]	f3. screening for infectious disease	f4. immunisation for all children	f5. referral and support for common infections	f6. management of infectious disease outbreaks	[WHO menu blank]
	g. non‐communicable diseases (incl. sensory function, physical disability, oral health, nutrition, and physical activity)	g1. Promotion of oral health care; reduced sugar; physical activity; appropriate sun exposure	g2. provision of nutrition and physical activity education	g3. screening for vision and/or hearing problems	g4. micronutrient supplementation	g5. referral and support for chronic conditions, disability, overweight, asthma	[WHO menu blank]	g7. support for policies on anaphylaxis
	h. mental health, substance use, and self‐harm	h1. mental health promotion (individual, group, whole of school)	h2. mental health education	h3. screening for mental health concerns	[WHO menu blank]	h5. support and referral for psychosocial issues, stress, mental health, behavioural, and emotional problems	[WHO menu blank]	h7. support for mental policies on mental health promotion and bullying prevention

Interpretative summary: This table has two dimensions: health area and type of activity undertaken. High frequency domains are shaded in green. Across Australia, the highest frequency activities were *support and referral for psychosocial assessment, stress, mental health, behavioural, and emotional problems*, followed by *promotion of care seeking and health literacy*. Medium frequency domains are shaded in orange, and low frequency domains in red.

### 3.10 Models of Nursing Service Delivery

Preliminary analysis identified a need to develop a new classification system for categorising models of nursing service delivery in Australian schools. Only one existing classification system, that developed by the Sax Institute (2021), was identified in the Australian literature. A plan to adopt this classification system was abandoned because the Sax Institute reported on an international data set, and the categorisations did not align well with the emerging Australian findings. In the newly developed classification system, models of nursing service delivery were categorised according to whether nurses were based in the school (full time, part time or unspecified hours) or visitors to the school (regularly or occasionally). In many cases, this information was not available, and the model of service delivery was categorised as unspecified. This resulted in a framework comprising six categories: (i) the nurse was based in the school and available on a full‐time basis, (ii) the nurse was based in the school and available on a part‐time basis, (iii) the nurse was based in the school but the hours were unspecified, (iv) the nurse was a regular visitor to the school, (v) the nurse was an occasional visitor to the school and (vi) the location and availability of the nurse was not specified or the source included a variety of models (Table 4). A nurse who was based in a school part‐time was differentiated from a nurse who was a regular visitor by characteristics such as whether they had a dedicated workspace at the school and whether they were integrated into the school staffing team. This approach is consistent with the WHO's (2021a) framework for school health services, which differentiates between school‐based and school‐linked delivery models based on provider location and level of organisational integration. This comprehensive categorisation enabled systematic analysis of the heterogeneous approaches to school nursing service provision encountered in the analysis.

**TABLE 4:** List of variables employed in frequency calculations.

Source type	Peer‐reviewed
	Not peer reviewed
Study type (peer‐reviewed sources)	Primary research – Qualitative
	Primary research – Quantitative
	Primary research – Mixed methods
	Primary research – Other
	Descriptive paper
	Discussion paper
Year of publication (range)	1970–1974
	1975–1979
	1980–1984
	1985–1989
	1990–1994
	1995–1999
	2000–2004
	2005–2009
	2010–2014
	2015–2019
	2020–2025
State	Queensland
	Victoria
	Western Australia
	New South Wales
	Tasmania
	Australian Capital Territory
	Northern Territory
	South Australia
	Unspecified/multiple
Service distribution	Urban
	Statewide
	Regional
	Urban and regional
	Urban, regional and remote
	Remote
	Unspecified
Education sector	Government
	Government and Non‐Government
	Non‐government
	Unspecified
School grade level	Primary
	Secondary
	Primary and Secondary
	Primary, Secondary and other
	Unspecified
Employer	Government
	General Practice
	School
	Various
	Unspecified
Models of nursing service delivery	the nurse was based in the school and available on a full‐time basis (‘in school – full‐time’)
	the nurse was based in the school and available on a part‐time basis (‘in school – part time’)
	the nurse was based in the school, but the hours were unspecified (‘in school – unspecified’)
	the nurse was a regular visitor to the school (‘visitor – regular’)
	the nurse was an occasional visitor to the school (‘visitor – occasional’)
	the location and availability of the nurse was not specified, or the source included a variety of the above (‘unspecified’)
Scope of nursing work	a1. promotion of care seeking and health literacy
	a2. support for health promoting curriculum, including delivery of health education in the classroom
	a3. school entry health check‐ups
	a5. provision of first aid; administration of medications; referral and support for minor illness
	a7. support for policies on health promotion, injury prevention and other aspects of HPS; training of school staff
	b1. promotion of appropriate use of electronic devices, adequate sleep, and parenting skills
	d5. support and referral for victims of violence (eg: child abuse, neglect)
	e5. support and referral for sexual health – contraceptive counselling; pregnancy; STIs; menstruation management
	f4. immunisation for all children
	g1. promotion of oral health care, reduced sugar, physical activity, and appropriate sun exposure
	g3. screening for vision and/or hearing problems
	g5. referral and support for chronic conditions, disability, overweight, asthma
	h1. mental health promotion (individual, group, whole of school)
	h5. support and referral for psychosocial issues, stress, mental health, behavioural, and emotional problems

### 3.11 Data Analysis

Extracted data was categorised in a Microsoft Excel document by the first author. The list of categories employed in the analysis is shown in Table 4. Variables required for frequency analysis were checked and amended to ensure consistent naming, and all categorisations underwent verification procedures to confirm the inclusion of all sources. Ten percent of all categorisations were randomly selected and independently verified by a second team member. Discrepancies were discussed and the process repeated until there was consistent agreement. Frequencies of variables were calculated using Microsoft Excel's integrated statistical functions. All calculations underwent verification procedures to ensure cumulative frequencies totalled 100%.

## 4 Results

### 4.1 Characteristics of the Included Sources

A complete list of the sources (*n* = 142) is provided in the Supplementary File. Most sources were peer‐reviewed (*n* = 81). The earliest source was from 1974, and the most recent from 2024, spanning 50 years. The volume of peer‐reviewed and non‐peer‐reviewed literature published since 1974 increased significantly over time, especially since 1995. Of the 81 peer‐reviewed papers, study designs included qualitative research (*n* = 28), quantitative research (*n* = 25), mixed methods (*n* = 9), descriptive papers (*n* = 16), and other designs (*n* = 3). The included sources were published in more than 70 unique journals. The education literature yielded few papers for inclusion, while many papers were published in medical journals with a specific clinical focus such as ophthalmology (in the case of vision screening) or immunisation. There were numerous occupational titles for nurses working in schools, with the most frequent occupational title being ‘school nurse’. The service with the greatest number of sources was the school‐based youth health nurse program in Queensland.

### 4.2 Sources by State/Territory, Service Distribution, Education Sector and School Type

The included sources described nursing work in every Australian state and territory. A descriptive summary of papers included in the review is shown in Table 5. Most sources were from Queensland (*n* = 30), New South Wales (*n* = 27), Victoria (*n* = 24) and Western Australia (*n* = 24), with fewer sources from South Australia (*n* = 8), Tasmania (*n* = 7), the Northern Territory (*n* = 5) and the Australian Capital Territory (*n* = 2). This is broadly representative of the population (Australian Bureau of Statistics 2025) and number of schools in each jurisdiction (Australian Curriculum, Assessment and Reporting Authority n.d.a). Some sources either did not specify the state or combined data from multiple states (*n* = 15). Service delivery was most frequently reported in urban areas (*n* = 38) or statewide (*n* = 35), with a high proportion of sources not specifying service distribution (*n* = 36). Only two sources (*n* = 2) described services in remote areas. Just under half the sources exclusively described services in government schools (*n* = 54), while 10 sources exclusively described services in non‐government schools. This too is broadly representative of the proportion of government and non‐government schools in Australia (Australian Curriculum, Assessment and Reporting Authority n.d.a). Forty‐nine sources did not specify in which education sector (government/non‐government) service delivery occurred. The highest reported frequency of services was in secondary schools (*n* = 63), followed by primary schools (*n* = 28) and primary and secondary schools combined (*n* = 24), as shown in Table 5. Secondary schools are overrepresented relative to the larger number of primary schools (Australian Curriculum, Assessment and Reporting Authority n.d.a) and primary school students in Australia (Australian Curriculum, Assessment and Reporting Authority n.d.b).

**TABLE 5:** Descriptive summary of sources included in the review.

Category	Variable	Count
Jurisdiction	Queensland	30
	New South Wales	27
	Victoria	24
	Western Australia	24
	Unspecified/multiple	15
	South Australia	8
	Tasmania	7
	Northern Territory	5
	Australian Capital Territory	2
	Unspecified/multiple	15
Location	Urban	38
	Unspecified	36
	Statewide	35
	Regional	20
	Urban & regional	8
	Urban, regional & remote	3
	Remote	2
Education sector	Government	54
	Unspecified	49
	Government & non‐Government	29
	Non‐government	10
School grade level	Secondary	63
	Primary	28
	Primary & secondary	24
	Unspecified	17
	Primary, Secondary & other	10
Employer	Government	85
	Unspecified	35
	Various	11
	School	10
	General Practice	1

### 4.3 Scope of Nursing Work

The included sources (*n* = 142) were categorised according to the WHO's menu of interventions for school health services, as shown in Table 3. Domains with high frequency are shaded in green, those with medium frequency in orange, and those with low frequency in red. All sources were categorised in at least one domain, and none of the sources described nursing work outside the scope of the WHO domains.

At a national level the highest reported frequency of nursing activity across all states and territories was *support and referral for psychosocial assessment, stress, mental health, behavioural, and emotional problems* (*n* = 52), followed by *promotion of care seeking and health literacy* (*n* = 41). Both focussed primarily on secondary school students, while the nursing activity with the highest frequency in the primary school context was *school entry health checkups* (*n* = 31). The lowest frequency activities across the data set were *support and referral for victims of violence* (*n* = 7), *promotion of appropriate use of electronic devices, adequate sleep, and parenting skills* (*n* = 3), and *promotion of oral health care, reduced sugar, physical activity and appropriate sun exposure* (*n* = 2). The WHO does not recommend that every country should implement every intervention (WHO 2021a), and several domains (shaded in red in Table 3) had a frequency count of zero.

Analysis at the state and territory level provides a more nuanced picture. A heat map of the frequency of nursing activity stratified by Australian jurisdiction is provided in Table 6, illustrating the significant variation in frequency of reported activities observed at the state and territory level. For example, Queensland and Victoria contributed significantly to the highest reported frequencies at the national level, while South Australia and the Northern Territory contributed minimally to these frequencies.

**TABLE 6:** Heat map of frequency of nursing activity by WHO (2021a) school health area in each Australian jurisdiction (government and non‐government combined).

Australian jurisdiction	WHO school health area													
	a1: Health promotion, care seeking	a2: Health curriculum	a3: School entry screening	a5: First aid, medications, minor illness	a7: Policy, training	b1: Parenting, sleep, electronic device use	d5: Victims of violence	e5: Sexual health, contraception, pregnancy, menstruation	f4: Immunisation	g1: Oral health care, physical activity, sun safety	g3: Vision, hearing screening	g5: Chronic conditions, disability, asthma	h1: Mental health promotion	h5: Psychosocial, mental health, behavioural
Queensland	12	10	3	3	8	0	2	9	1	1	6	8	3	10
Victoria	7	4	7	7	2	3	1	2	4	0	1	5	5	11
Western Australia	3	5	7	2	2	0	2	3	3	0	4	4	6	12
New South Wales	4	3	7	5	3	0	0	2	4	1	5	2	0	4
Tasmania	7	2	1	2	1	0	0	4	0	0	4	5	1	5
Australian Capital Territory	1	0	1	1	0	0	0	1	0	0	0	1	0	1
Northern Territory	2	2	1	1	1	0	2	1	1	0	1	2	0	2
South Australia	1	0	3	1	1	0	0	1	2	0	1	1	2	1
Unspecified/multiple	4	3	1	4	1	0	0	2	2	0	4	8	4	6
Total	41	29	31	26	19	3	7	25	17	2	26	36	21	52

Interpretative summary: This Table illustrates the variation in frequency of reported nursing activity by jurisdiction. Queensland and Victoria had higher reported frequencies across more health areas (shaded in green). South Australia and the Northern Territory had more health areas shaded orange and red, indicating less frequently reported activities.

In line with the review question, the included sources were also analysed by employer. As detailed in Table 5, more than half the sources described the work of nurses employed by their respective state or territory government (*n* = 85), while the number of sources where nurses were employed directly by their school was comparatively low (*n* = 10). This prohibited a robust comparison of the scope of nursing work undertaken by nurses employed by the government or by their school. The available data suggest that nurses employed by their school are proportionally more likely to be engaged in the provision of first aid, administration of medications and referral and support for minor illness than their government counterparts. In contrast, the most frequently occurring activities for nurses employed by the government were *support and referral for psychosocial assessment, stress, mental health, behavioural and emotional problems* (*n* = 33), followed by *promotion of care seeking and health literacy* (*n* = 31). Significant variation was observed between states and territories.

### 4.4 Models of Nursing Service Delivery

Models of nursing service delivery were categorised according to whether the nurse was based in the school (full time, part time or unspecified hours) or a visitor to the school (regularly or occasionally). Each source was categorised to one model of service delivery but was frequently unspecified (*n* = 60). The most frequently identified model was a nurse based in the school with unspecified hours of availability (*n* = 44). This model was especially common in Queensland (*n* = 14) and Western Australia (*n* = 13). The frequency of a nurse based in the school on an explicitly full‐time basis was comparatively rare (*n* = 5).

**FIGURE 2:**
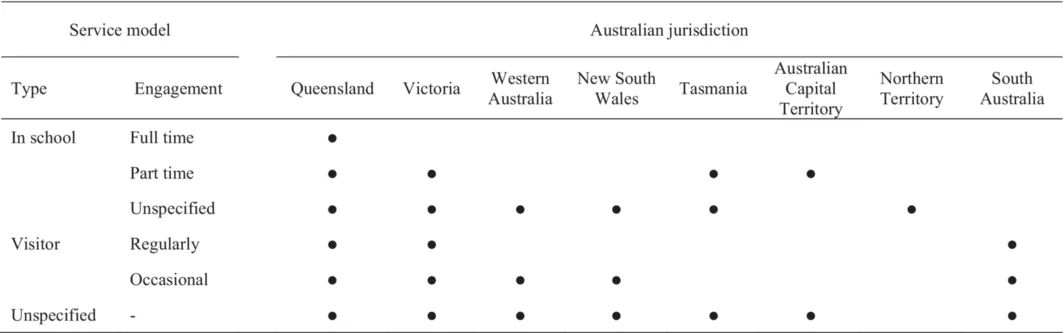
Matrix chart depicting government model of school nursing service delivery in each Australian jurisdiction. Unspecified (*n* = 1) is not included. Non‐government schools (*n* = 10) excluded.

The included sources were also analysed by whether the nurse was employed by the school or the government. Ten sources described models of service delivery where the nurse was employed by the school; all 10 were school‐based models of care. The most reported model of service delivery when the nurse was employed by the government was also school‐based; however, there was significant variation between Australian jurisdictions, as illustrated by the matrix chart in Figure 2. The data suggests that some Australian jurisdictions – such as Queensland – are more likely to employ nurses in a wider range of service delivery models than other states or territories.

## 5 Discussion

The aim of this scoping review was to map the scope of nursing work and models of service delivery in Australian primary and secondary schools for children aged 3–18 years. There are two key findings. Our first finding is the significant jurisdictional variation at the level of the states and territories, across education sectors and between employers. This contributes to inequitable access to nursing services at school and reflects the absence of a coherent national approach to service delivery, policy, and workforce planning. Our second finding is that the same evidence, when viewed from a national perspective, indicates that the scope of nursing work undertaken in Australian schools is extensive, and models of service delivery are flexible and varied. Taken together, these findings suggest a rich and largely unacknowledged history of nursing work in Australian schools, but also untapped potential to strengthen nursing service delivery for improved health and developmental outcomes across a wider population of school‐attending children and young people. Our findings will be of interest to a wide range of stakeholders, including governments, schools, parents, health and education policy makers, school nursing researchers and school nurses themselves. We first examine why our review is important internationally for its methodological innovation before exploring the implications at the national level.

To the best of our knowledge, this is the first study worldwide to categorise school nursing activities in a specific country using the WHO's school health services menu of interventions, highlighting its international significance. Our study demonstrates the feasibility of this approach for categorising nursing work in schools. This is important because school nursing practice varies between countries (Baltag et al. 2015), and cross‐national comparisons have been complicated owing to the absence of a suitable framework for analysis. The lack of comparable studies prevents us from benchmarking our findings against other countries; however, we encourage school nursing researchers internationally to conduct similar studies to facilitate cross‐national comparisons in the future.

Our study is also important at the national level. Prior to our study, the state of the evidence was unclear, presenting a significant barrier for researchers and policy makers attempting to develop a coherent understanding of Australian school nursing and hindering the development of future research in the field. Our rigorous search for literature describing nursing work in Australian schools identified a larger quantity of literature than expected, spanning 50 years. Importantly, we did not identify any previous scoping reviews or systematic syntheses. In other nursing specialties, 50 years of literature would suggest a mature field of practice with a solid evidence base; however, much of the literature we identified was hidden, rendering the evidence base fragmented rather than cumulative. The systematic comprehensive synthesis reported in this review has therefore addressed an important knowledge gap.

Having overcome the issue of fragmented evidence, our systematic synthesis of the literature enabled us to identify patterns that were previously obscured. As shown in Table 5, we first examined the jurisdictional distribution of the evidence and identified that almost 90% of the included sources related to a specific state or territory; sources with a national focus were uncommon. Most sources described government school nursing services. We also observed a greater frequency of evidence for nurses working in secondary schools despite primary school students being more numerous (Australian Curriculum, Assessment and Reporting Authority n.d.b). Further analysis provided more nuanced insights and highlighted significant jurisdictional variation in the scope of nursing work delivered, especially at the level of the states and territories. The WHO's menu of interventions explicitly encourages the selection of school health services oriented to a country's identified health needs (WHO 2021a), however, the level of variation identified in our review cannot be explained with reference to resource availability or population health needs. For example, the data shown in Table 6 provides little evidence of nursing services in South Australian government schools and a wide range of nursing services in Queensland government schools. Although included papers occasionally gave insight into factors that may have shaped jurisdictional variation such as state government election commitments (Millward 2017), withdrawal of existing funding (Queensland Nurses' Union of Employees 2013), and isolated federal government grants (Collard 1994), an analysis of these factors was beyond the scope of our review.

The jurisdictional variation in the scope of nursing work delivered was also observed in models of service delivery: schools in some jurisdictions had significantly greater access to nursing services than other jurisdictions. Taken together, these findings are difficult to justify and raise critical questions about equity of access to school‐based nursing support for children and young people. Equity of access is a fundamental principle of social justice and a cornerstone of the Australian health system (Australian Government Department of Health, Disability and Ageing 2025), yet the variation documented in our review means that Australian children with identical health needs receive vastly different levels of school‐based nursing services in consequence of arbitrary factors such as state borders. As illustrated in Table 6, children attending schools with well‐resourced school nursing services may benefit from early identification of health concerns, management of chronic conditions, health promotion initiatives, and support to access specialised health services. In contrast, students attending schools with limited access to nursing service provision may be at greater risk of delayed identification of health and developmental needs, and consequently, reduced health outcomes and educational attainment (WHO 2021a). These disparities are particularly concerning in the wider context of primary health care for children. General practitioners are the first line of primary health care in Australia, yet child attendance is falling, especially in non‐metropolitan areas and among families with low socioeconomic status (Ou et al. 2017). Schools are therefore a unique point of universal contact with children and young people, including those from disadvantaged or hard‐to‐reach populations who may not regularly access primary health care services. For example, children living in remote and rural areas who typically experience poorer health and developmental outcomes than their urban counterparts (Sanford et al. 2021), and Aboriginal and Torres Strait Islander children, who experience higher rates of developmental delays, chronic disease and social and emotional wellbeing challenges (Australian Institute of Health and Welfare 2024b). School nurses are well positioned to identify and address the social determinants of health that contribute to these health disparities (Bowen 2023) but coordinated efforts to improve access to school‐based nursing services for all Australian children will require work across jurisdictions and sectors. The alternative risks perpetuating the inequitable access to school nursing services identified in our review.

While children and young people are the primary recipients of school nursing services, our findings are also relevant to schools and parents. These stakeholder groups bear the consequences of jurisdictional variation in scope and availability of nursing services, primarily because school staff are commonly tasked with responding to children's health concerns in Australian schools. Despite having limited training for the role, school staff such as teachers and teacher assistants regularly administer student medications, manage chronic conditions including diabetes and epilepsy and respond to acute life‐threatening emergencies such as anaphylaxis (Rossi et al. 2016). School staff report feeling overloaded by student health demands, citing the heavy responsibility and emotional burden (McCuaig et al. 2019). For parents of children with health needs, concern about the adequacy of the current system prompts some parents to forego employment to remain available to their child's school, while others choose a school with a registered nurse on staff (Marks et al. 2018). While primary responsibility for monitoring health service provision by school staff rests with the principal, insight into these activities does not appear to be in the public domain, and whether the health needs of school students are being adequately addressed has not been investigated. Developing a more coherent and nationally consistent approach to nursing services in schools could enhance health outcomes for Australian children and young people, while simultaneously supporting schools and families to manage increasingly complex student health needs.

Paradoxically, while the jurisdictional variations identified in our review are concerning, the same evidence highlights the adaptable and multifaceted strengths of nursing work undertaken in Australian schools over the past 50 years. Categorising nursing activities according to the WHO's menu of interventions for school health services as shown in Table 3 provides evidence that collectively, nurses working in Australian schools have historically delivered a wide range of services consistent with the WHO definition of a comprehensive school health service (2021a). The WHO defines a comprehensive school health service as one that typically addresses at least four but ideally all of the following health areas: positive health and development; unintentional injury; violence; sexual and reproductive health; communicable disease; non‐communicable disease; sensory functions; physical disability; oral health; nutrition and physical activity; and mental health, substance use and self‐harm. Importantly, the Australian national standards for school nurses also endorse this breadth of nursing activity (Australian Nursing and Midwifery Federation 2019). The extent to which services in our data set provided a comprehensive school health service aligned with the WHO definition was beyond the scope of our analysis. However, Table 3 suggests that nurses are well‐positioned to deliver these services, and a consistent approach to nursing service delivery in schools would better leverage this capability.

Although our findings revealed a breadth of nursing activity across Australian schools, we also identified gaps. The strongest gap appears to be nursing engagement in the delivery of health education. Australia has a comprehensive national health and physical education curriculum (Australian Curriculum, n.d.). In primary schools, health is commonly taught by generalist teachers who may rely heavily on their own personal understanding and values related to health (Cruickshank et al. 2022). Secondary school teachers may be more likely to possess specialist health education, but understanding and assessing the reliability of health information and its application in diverse contexts has been identified by some teachers as challenging (Peralta et al. 2022). Australian registered nurses undergo explicit professional preparation for assessing the reliability of health information for application in diverse contexts. The lack of evidence for their engagement in the delivery of health education, even as co‐facilitators with teachers, is therefore concerning. Table 3 also suggests that nurses are poorly engaged to assist students who are victims of violence, in reducing use of electronic devices, sugar intake, and sun exposure, and to improve sleep, parenting skills, oral health and physical activity. These are contemporary health issues among Australian children and young people, and a failure to use school nursing services to address these issues is surprising considering the priority they are given in national frameworks and strategies for children and youth (Australian Government Department of Health 2019; Australian Research Alliance for Children and Youth 2014).

Addressing the jurisdictional variation and gaps identified in our review will require coordinated action across jurisdictions and sectors, with implications for nursing education, workforce planning and government funding and policy. Fortunately, this important work is already underway in the form of the recently published federal government report ‘Unleashing the potential of our workforce’ (Cormack 2024). While not specific to nursing work in schools, recommendations of relevance to our review include governments working together to review the barriers that prevent nurses from working to their full scope, and addressing education opportunities, funding mechanisms and regulatory issues. While all of these are important, coordinated education and workforce planning are central to strengthening school nursing service delivery in Australia. The lack of a credentialled pathway or recognised postgraduate qualification for school nursing in Australia perpetuates the phenomenon of the ‘accidental school nurse’, where registered nurses become school nurses by accident, not design (Moyes and Karemba 2025). The absence of nationally coordinated workforce planning prompts each nurse, school or government health service to design its own version of the role, entrenching disparate practice. Foundational education for working in a school does not infer that every school nurse should undertake the same role; there is ample room for sub‐specialities within a strengthened approach to school nursing. But the absence of foundational education contributes to poor stakeholder understanding of school nursing practice and has consequences for school nursing professional identity (Moyes and Hayward 2025). In the absence of a common foundation, the speciality will struggle to articulate its value and scope to policymakers and schools, leaving it vulnerable to continued fragmentation and marginalisation. This is demonstrated by policy responses such as adding other health disciplines into schools on an ad hoc basis before properly supporting existing school nurses to practice to their full scope. For example, the AUD$43 million program placing doctors into 100 secondary schools in Victoria (State of Victoria 2021, 2024) has provided little guidance as to how existing government secondary school nurses should collaborate with doctors in schools, other than to ‘work together … and support the work of both services’ (Department of Education 2019, 9). This approach also neglects to capitalise on the skills and expertise of the existing school nursing workforce by offering enhanced career pathways towards advanced practice roles. Nurse practitioners are commonly employed in school‐based health centres in the United States (Borkowski et al. 2023), and the importance of this advanced practice role has been acknowledged by the Australian Government (Australian Government Department of Health and Aged Care 2023). Exploring these models could deliver dual benefits: strengthening service delivery at a lower cost to the taxpayer than the employment of doctors and enhancing the career pathway for Australian school nurses.

By mapping the school nursing landscape for the first time, our review establishes the evidence base for strengthening school nursing service delivery in Australia. A reimagined approach should be one that acknowledges the rich history of nursing work in schools, recognises and builds upon the existing workforce, and values the expertise nurses bring to schools. A nationally coordinated, systematic approach could help ensure more equitable access to school‐based nursing services for all Australian children and young people.

## 6 Strengths and Limitations

A strength of the scoping review was the author team, representing five of Australia's eight states and territories, all of whom had previous or current experience as school nurses in Australia and four of whom had published in the academic school nursing literature. The search strategy was robust, following an accepted review strategy, and the protocol was registered (see Open Science Framework https://osf.io/6yqrm) and published (see https://doi.org/10.11124/JBIES‐24‐00151) before undertaking the review.

Our findings also have some limitations. Firstly, despite the large data set, our findings may reflect a lack of research rather than a lack of nursing activity. Additionally, while the volume of research increased over time, our findings may not be representative of current‐day nursing work in schools if current practices have not been published, even in the grey literature. For example, children receiving education in special schools require specialised nursing care to support their complex health needs (Singer 2012), yet we observed a paucity of literature exploring the work of nurses in schools for children with disabilities (Hayward 2024). We also observed a lack of evidence for the work of enrolled nurses in schools. Enrolled nurses work under the direction of a registered nurse but are responsible for their own practice (Australian Nursing and Midwifery Accreditation Council 2016). Given their presence in schools (Government of Western Australia 2025), this absence from the literature reflects an underrepresentation in research that fails to capture the full spectrum of nursing work in Australian schools. Finally, there is a paucity of papers describing the work of nurses employed directly by a school, despite evidence of their presence (Moyes and Karemba 2025). This neglect prevented robust analysis of their professional contributions for this review.

The second limitation is that our analysis was complicated by inconsistent reporting practices. While reviewing 50 years of literature might reasonably present challenges, in the future these issues could be significantly reduced by adopting consistent reporting frameworks to enable better service evaluation and more meaningful comparisons across jurisdictions.

Finally, some relevant sources may not have included the applied keywords and therefore could have been excluded. Further, the combination of keywords ‘school’ and ‘nurs*’ provided a great many articles related to ‘schools of nursing’ in universities and colleges, making the task of screening more cumbersome. However, there was no way to avoid these primary keywords, and their use did ensure that all appropriate literature was considered.

## 7 Future School Nursing Research

Future research should prioritise several key directions, including outcomes and cost‐effectiveness analyses. The former is essential for examining the impact of nursing work in schools on health and educational outcomes, and the latter for determining economic value and informing resource allocation. Comparative studies would be especially useful given the likely cost differences across different nursing service delivery models identified, and the renewed focus on student wellbeing in the Australian Government's *Better and Fairer* schools funding agreement (Australian Government Department of Education 2024).

Our study categorised school nursing activities according to the WHO (2021a) menu of school health interventions and demonstrated that this was a feasible approach for systematically documenting the breadth of school nursing practice. This method should be considered by future researchers to standardise the reporting of nursing work in schools for improved international comparison. We also encourage Australian school nursing researchers to adopt our categorisation of school nursing service delivery for greater standardisation of the Australian school nursing research. Researchers should specify a minimum data set including the setting in which the service is delivered (government or non‐government school) and the employer. More descriptions of school nursing in South Australia, Tasmania, the Northern Territory, and the Australian Capital Territory are required, as is school nursing research in non‐urban areas and non‐government and primary schools. Research investigating the work of enrolled nurses and nurses employed by their school should also be prioritised.

## 8 Conclusions

The presence of nurses in Australian schools stretches back decades, but prior to our review there was limited clarity about the scope of nursing work undertaken or the service delivery models in use. This scoping review sought to map the scope of nursing work and models of service delivery in Australian primary and secondary schools for children aged 3–18 years. We identified that Australia has a rich history of nursing work in schools, with nurses collectively delivering a wide range of services consistent with the WHO's definition of comprehensive school health services. Models of nursing service delivery are both varied and flexible. We also identified significant jurisdictional variation: Australian children, parents and schools have access to vastly different levels of school nursing services that are difficult to justify. A national approach would ensure equitable access to school‐based nursing services for all Australian children and young people at a time when schools are increasingly accommodating students with complex health needs.

## Author Contributions

A.M. conceptualised the review design. C.W., E.R., B.A.H. and A.M. screened the studies. I.H., E.‐J.P., E.R., C.W. and A.M. extracted data. A.M. compiled the results and wrote the first draft. A.M., B.H. and C.W. refined the draft. All authors reviewed the final draft of the paper.

## Funding

The authors have nothing to report.

## Ethics Statement

This is a scoping review of existing literature and ethics approval was not required.

## Conflicts of Interest

The authors declare no conflicts of interest.

## Supporting information


**Data S1:** jan70562‐sup‐0001‐DataS1.xlsx.

## Data Availability

The data that supports the findings of this study are available in the Supporting Information of this article.
